# In Vitro ^31^P MR Chemical Shifts of In Vivo-Detectable Metabolites at 3T as a Basis Set for a Pilot Evaluation of Skeletal Muscle and Liver ^31^P Spectra with LCModel Software

**DOI:** 10.3390/molecules26247571

**Published:** 2021-12-14

**Authors:** Petr Sedivy, Tereza Dusilova, Milan Hajek, Martin Burian, Martin Krššák, Monika Dezortova

**Affiliations:** 1 MR-Unit, Department of Diagnostic and Interventional Radiology, Institute for Clinical and Experimental Medicine, Videnska 1958/9, 140 21 Prague, Czech Republic; petr.sedivy@ikem.cz (P.S.); tereza.dusilova@ikem.cz (T.D.); milan.hajek@ikem.cz (M.H.); martin.burian@ikem.cz (M.B.); 2 Division of Endocrinology and Metabolism, Department of Internal Medicine III, Medical University of Vienna, 1090 Vienna, Austria; martin.krssak@meduniwien.ac.at; 3 High-Field MR Center, Department of Biomedical Imaging and Image-Guided Therapy, Medical University of Vienna, 1090 Vienna, Austria

**Keywords:** in vivo MR spectroscopy, ^31^P MRS, LCModel, liver, muscle

## Abstract

Most in vivo ^31^P MR studies are realized on 3T MR systems that provide sufficient signal intensity for prominent phosphorus metabolites. The identification of these metabolites in the in vivo spectra is performed by comparing their chemical shifts with the chemical shifts measured in vitro on high-field NMR spectrometers. To approach in vivo conditions at 3T, a set of phantoms with defined metabolite solutions were measured in a 3T whole-body MR system at 7.0 and 7.5 pH, at 37 °C. A free induction decay (FID) sequence with and without ^1^H decoupling was used. Chemical shifts were obtained of phosphoenolpyruvate (PEP), phosphatidylcholine (PtdC), phosphocholine (PC), phosphoethanolamine (PE), glycerophosphocholine (GPC), glycerophosphoetanolamine (GPE), uridine diphosphoglucose (UDPG), glucose-6-phosphate (G6P), glucose-1-phosphate (G1P), 2,3-diphosphoglycerate (2,3-DPG), nicotinamide adenine dinucleotide (NADH and NAD^+^), phosphocreatine (PCr), adenosine triphosphate (ATP), adenosine diphosphate (ADP), and inorganic phosphate (Pi). The measured chemical shifts were used to construct a basis set of ^31^P MR spectra for the evaluation of ^31^P in vivo spectra of muscle and the liver using LCModel software (linear combination model). Prior knowledge was successfully employed in the analysis of previously acquired in vivo data.

## 1. Introduction

Magnetic resonance spectroscopy (MRS) equipment and protocols have become a standard part of clinical MR systems; however, the interest is mostly focused on proton (^1^H) in vivo MR spectroscopy, which is used in many research and clinical applications.

Another spectroscopy technique is phosphorous (^31^P) MRS, which has an even longer history of in vivo applications [1]. Despite lower sensitivity, there is no overwhelming prominent signal in ^31^P MR spectra that would require a special suppression technique (as does the signal of water in ^1^H MR spectroscopy). In addition, ^31^P MR signals of three important compounds of energy metabolism—phosphocreatine (PCr), adenosine triphosphate (ATP), and inorganic phosphate (Pi)—show relatively high concentrations and are well-resolved in in vivo ^31^P MR spectra. Moreover, ^31^P MR spectra can be measured repeatedly with rapid succession during a physical challenge and reveal rapid changes in energy metabolites (and, e.g., pH) over time. This is dynamic ^31^P MRS [2]. This technique thus significantly contributes to the understanding of energy metabolism of muscles and other tissues [3].

**Figure 1:**
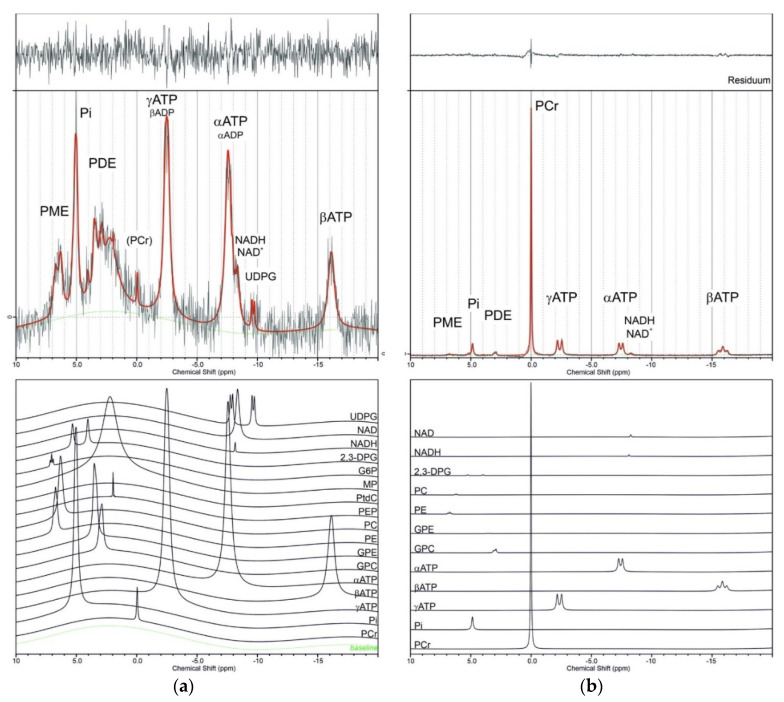
Examples of in vivo ^31^P spectra of the liver (**a**) and calf muscles (**b**). Black lines represent measured spectra, red lines show spectra calculated by LCModel with the application of the BASIS^p^ data set, and the green line represents the calculated baseline. In the upper panels of the picture, individual contributions of metabolites to the calculated spectra are shown: UDPG—uridine-diphosphoglucose; NADH/NAD^+^—nicotinamide adenine dinucleotide; 2,3-DPG—2,3 diphosphoglycerate; G6P—glucose-6-phosphate; G1P—glucose-1-phosphate; MP—membrane phospholipids; PEP—phosphoenolpyruvate; PC—phosphocholine; PE—phosphoethanolamine; GPE—glycerophosphoethanolamine; GPC—glycerophosphocholine; PtdC—phosphatidylcholine (overlapped with PEP); ATP—adenosine triphosphate; ADP—adenosine diphosphate; Pi—inorganic phosphate; PCr—phosphocreatine (in the case of the liver spectrum, this signal is the contamination; it can serve as the standard for chemical shift measurement); PME—phosphomonoesters region; PDE—phosphodiesters region. (In vivo spectra of the liver and calf muscles of a young, healthy volunteer were taken from the database of the MR group IKEM. The liver spectrum was measured at 3 T TRIO MR system, 1 D ISIS, TR = 2 s, acq = 196, TE = 0.2 ms, bandwidth 3000 Hz; muscle spectrum was obtained at VIDA 3 T MR system, FID sequence, TR = 15 s, bandwidth 2000 Hz; unpublished results.

Most of the ^31^P MR studies have been performed at 1.5 and 3 T systems, which provide sufficient signal for dynamic studies and are available for routine clinical examinations and research. In addition to well-resolved signals, such as PCr, Pi, and ATP in muscles, there are a number of metabolites that resonate in the narrow range between 2–8 ppm and that overlap each other and arise primarily from inorganic phosphate (Pi), phosphomonoesters (PME), and phosphodiesters (PDE) (see Figure 1). The identification of these metabolites in in vivo spectra is performed by comparing their respective chemical shifts with the chemical shifts of individual solutions of chemical compounds measured in vitro.

Recently, a few ideas have been suggested that have sparked new research and different interpretations. First, in the liver, the potential signals of phosphoenolpyruvate (PEP), a key component of glycolysis and gluconeogenesis, and phosphatidylcholine (PtdC) both resonate at a similar position around 2 ppm [4,5,6]. Even though PtdC is a major bile component, it could also contribute to the signal from the hepatic parenchyma, and its contribution cannot be neglected [4].

Second, applying proton decoupling at 3 T, or using ultra-high-field systems (≥7 T), the resonances of PME and PDE can easily be split into individual components of phosphocholine (PC), phosphoethanolamine (PE), glycerophosphocholine (GPC), and glycerophosphoethanolamine (GPE). Total PDEs and/or PMEs are linked to glucose homeostasis [7], overall skeletal muscle health [8], and liver cirrhosis [9]. Lately, specific levels of skeletal muscle GPC have been suggested as a biomarker for tissue-specific thyroid action [10].

Furthermore, the role of nicotinamide adenine dinucleotide (NADH) and uridine diphosphoglucose (UDPG) in energy metabolism is undisputed, and both are accessible to ^31^P MRS. Although their resonances are often unresolved (due to bad shim) or partially overlapped by the dominant αATP signal, the application of ^1^H-^31^P broadband decoupling can significantly improve their resolution even at 3 or 4 T magnetic fields (for details see Discussion, Section 3.4 and Section 3.5).

Another interesting task is the assignment of the second Pi skeletal muscle signal in several pathophysiological conditions [8,11,12,13]. Not only was there a significant increase observed in athletes [13] and a decrease in an obese sedentary population [11] but changes in patients with severe diabetes-related ischemia were also observed [8]. It was suggested that this signal arises from interstitia, but mitochondrial phosphate cannot be excluded. Even at 3 T, magnetic field signals in the range of 2 to 8 ppm are not well-resolved, and identification of metabolites in this range is based only on the comparison of signal maxima with in vitro data.

In this report, ^31^P chemical shifts and coupling constants are presented for fifteen metabolites that can be observed in living tissue using ^31^P MR spectroscopy. These MR chemical shifts were determined from the measurement of individual phantom solutions in a whole-body 3 T MR system, which mimicked standard in vivo conditions (temperature 37 °C and pH~7), thus enabling a comparison of these data to the results of high-field NMR spectroscopy and other literature data. Furthermore, we used these spectra acquired in phantoms to create a 3 T spectral basis, and we included some additional literature data that can be used for deconvolution of in vivo ^31^P MR liver and skeletal muscle spectra by LCModel software [14] (linear combination of individual components in the frequency domain). Creating this prior knowledge allowed an approach to the analysis of previously acquired in vivo spectra.

## 2. Results

All metabolites were measured in a solution of pH 7.0 and 7.5, which covered a range of cytosolic, interstitial, vascular, and mitochondrial pH values. Table 1 summarizes the concentrations, multiplicity, chemical shifts, and interaction constants (J-coupling constant) of all 15 compounds in phantoms as prepared and measured in this study. PCr was used as an internal standard with a chemical shift of 0.0 ppm. See spectra in Supplementary Material.

Solutions of metabolites were prepared in K_2_HPO_4_ and NaH_2_PO_4_ buffers, which guaranteed sufficient stability of the compounds for ^31^P MRS experiments within a few days.

Measured ^31^P chemical shifts of all compounds were used for the construction of a basis set for the evaluation of skeletal muscles and liver spectra, which is necessary for LCModel application. Figure 1 shows representative spectra of calf muscles and liver calculated with the newly constructed BASIS^p^. In addition to the basis set proposed by Deelchand [15], signals of UDPG, G1P, G6P, PEP, and PtdC were appended. Some chemical shifts were optimized during testing to better suit in vivo conditions. Additionally, signals of macromolecule membrane phospholipids (MP) and glycerophosphoethanolamine were included based on the literature data [15,16]. Finally, BASIS^p^ used for the analyses contained 19 signals of 17 model spectra summarized in Table 1. It was generated internally during analysis based on the specified input parameters. ADP was omitted intentionally as the α- and β-ADP resonances are overlapped by stronger α and γ-ATP resonances. The signals of the three ATP phosphorus signals covering a significant part of the spectral range (from −2 to −17 ppm) were described by three separate data sets to accommodate different linewidths (Lorentzian signals). The chemical shifts of α, β, and γ phosphorus atoms of ATP were also modified to better suit in vivo conditions.

In addition to the representative spectra of the liver and calf muscles (Figure 1), a pilot comparison of LCModel and jMRUI calculation of signal intensities of five randomly chosen liver spectra and five muscle spectra from our database was used. Coefficients of variation were used for the comparison, and results are summarized in Table 2.

## 3. Discussion

The availability of ultra-high field imagers (≥3 T) also enables the study of less prominent ^31^P metabolites in muscles [8,11,17,18], liver [4,9,19], and brain tissue [16,20,21]. These less frequently assessed metabolites may be divided into phosphomonoester (PME) and phosphodiester (PDE) groups according to their chemical shifts ranging from approx. 5–7 and 1–5 ppm, respectively. In the PME area, signals of chemical components, such as phosphoethanolamine (PE), phosphocholine (PC), glucose-1-phosphate (G1P), and glucose-6-phosphate (G6P), can be found. In the PDE area, signals of glycerophosphoethanolamine (GPE), glycerophosphocholine (GPC), phosphatidylcholine (PtdC), and phosphoenolpyruvate (PEP) can be recognized. Another area of interest is the chemical shifts from −7 to −8.5 ppm where, e.g., signals of αATP, adenosine diphosphate (αADP), nicotinamide adenine dinucleotide (NADH/NAD^+^), and uridine-diphosphoglucose (UDPG) can be found. The contribution of membrane phospholipids should also be taken into consideration [16].

The exact in vivo chemical shift position of ^31^P metabolites may be influenced by a number of parameters. One of the most important is pH, which differs in different compartments of cells and tissues or may change under pathologic conditions. Physiological pH of body tissues usually ranges from 7.0 to 7.5. Intracellular cytosolic pH 7.0 is the same as the pH of the endoplasmic reticulum [22,23,24] (hepatocytes); the interstitial pH of the brain cortex is 7.2 [25]; and the mitochondrial matrix ranges between pH 7.5 and 8.2 in various cells and their metabolic state [26,27]. Arterial and venous pH is about 7.3–7.45, and the pH inside red cells is slightly lower, about 7.2–7.3 [28,29]. However, tissue pH may reach lower values in ischemia or after anaerobic exercise in muscle. In the brain cortex, one hour of ischemia decreases interstitial and intracellular pH from 7.24 and 7.01 to 6.43 and 6.86, respectively [25]. The pH of a resting myocyte is about 7.0, which can increase slightly during exercise (about 0.1); however, during intensive (anaerobic) exertion, the pH drops from 7.0 to 6.5 or less [17].

Most of the molecules relevant to ^31^P MRS evaluation are located in the cell cytosol; therefore, at a pH of 7 under physiological conditions. NAD^+^/NADH, ADP, and ATP are also present in the mitochondrial matrix (pH 7.5–8). G6P is transported from the cytosol to the endoplasmatic reticulum (ER) to be dephosphorylated to glucose only in the hepatocytes. However, a typical pH of the hepatocyte cytosol and the ER is the same and equals pH 7 [22,23,24]. A prominent ^31^P signal of blood is 2,3-diphosphoglycerate (2,3-DPG), located in red cells, therefore with a pH of 7.2. However, in red cells, the 2,3-DPG signal is not only affected by pH but also modified by its binding to hemoglobin.

In the sections that follow, further individual metabolites are discussed in detail.

### 3.1. Energy Metabolites—Phosphocreatine (PCr), Inorganic Phosphate (Pi), Adenosinetriphosphate (ATP), and Adenosinediphosphate (ADP)

PCr, Pi, and ATP metabolites are closely related to energy turnover and come into prominence especially in muscle metabolism studies. PCr is a dominant signal in muscles, whereas, in the liver and kidney, it is not detected. In skeletal muscles, PCr concentration is about 33 mM and Pi is about 4.5 mM [30]. The PCr signal, with a chemical shift of 0.0 ppm, is widely used as an internal standard for in vivo ^31^P MR experiments; in our case, it was also used as an internal reference for all our phantoms. To verify the stability of PCr frequency with pH alterations in phantoms, the signal of two phantoms with a pH of 7.0 and 7.5 were acquired in one spectrum. In the resultant spectrum, only one PCr peak was then present, which implies an identical chemical PCr shift in both conditions. However, the chemical shift position of PCr is not totally insensitive to pH change if pH changes an order of several units, as McDowell and Stewart have shown [31]. For more details, see the literature, e.g., [17,32,33,34].

Pi, which participates in energy reactions in living tissue, has been present in all phantoms as a buffering agent. The chemical shift of Pi strongly depends on pH and is controlled by the equilibrium

$${\mathrm{HPO}}_{4}^{2-}+H^{+}⇆H_{2}{\mathrm{PO}}_{4}^{-}$$

Frequently used equation for the calculation of pH in vivo is based on the Henderson–Hasselbalch equation in the form:

pH = 6.75 + log ((δ − 3.27)/(5.63-δ))

where δ is the chemical shift frequency difference between pH-dependent Pi and pH-independent reference peak (in ppm). Usually, this reference peak is PCr, but it can also be, e.g., αATP [34] or some other metabolites [32]. In our phantoms, the δ was used to control the pH measured by a pH meter. In vivo, Pi is usually visible as a single signal, but under some pathological conditions or with improved spectral resolution due to the higher field-strength, an additional Pi signal can be visible [8,11,12].

The ^31^P MR spectrum of ATP is characterized by three phosphorus signals split into doublets (α, γ) and a triplet (β) due to J_P-H_. In vivo chemical shifts of ATP signals are significantly influenced by pH and the presence of ions, as are Zn, Cu, Mg^2+^, and Ca^2+^, which form ATP complexes with different chemical shifts. Depending on pH, ion concentration chemical shifts can be changed. The literature data show in vivo chemical shifts in the ranges: α~7–8, β ~16, and ~3–4 ppm [35,36]. It should be mentioned that only the β-ATP signal arises from pure ATP, whereas αATP includes small contributions from NAD^+^/NADH and αADP, and γATP usually contains a contribution from β-ADP.

In our phantoms, the chemical shifts of α and γ phosphorus are in this in vivo range; however, β-phosphorus is shifted up to 18.8 ppm. Nevertheless, this is in agreement with other findings reported in the literature [37,38,39,40].

Whereas ATP cellular concentration in muscles is assumed to be constant in young healthy individuals and is about 5.5 mmol/kg wet weight, which equals 8.3 mM cell water, ADP concentration is very low. Indeed, in resting muscle, it is only about 13 µmol [30], and thus impossible to detect by ^31^P MRS directly. For more information on ATP detection, see the literature [30,41,42,43].

### 3.2. Phosphomonoesters (PME)

Phosphomonoesters mainly represent intermediates on the phospholipid biosynthesis pathway. The main components of the PME signal are phosphocholine (PC) and phosphoethanolamine (PE), and small signal contributions are derived from sugar phosphates. PME resonate in a rather broad frequency range between 2 to 8 ppm, and thus they partly overlap with the PDE frequency region.

#### 3.2.1. Phosphocholine (PC) and Phosphoethanolamine (PE)

Both phosphocholine (PC) and phosphoethanolamine (PE) molecules have one phosphorous atom that forms a multiple triplet structure between 6 and 7 ppm. ^1^H decoupling simplifies the signal shape from a triplet to a singlet. The change of chemical shift is only 0.1–0.2 ppm between pH 7.0 and 7.5. The PE chemical shift of 6.7–6.8 ppm in the phantom was in good agreement with the in vivo value (6.78 ppm), and the chemical shift of PC (6.2–6.4 ppm) was slightly higher compared to the in vivo chemical shift of 5.9 ppm [36].

In ^31^P MRS, signals of PC and PE often overlap; therefore, they are usually evaluated as a combined PME (phosphomonoester) signal. An increase of PC was shown using high-resolution ^1^H MRS, and similarly, an increased PME signal was observed in vivo in various cancers; for review, see [44]. In muscles, PME concentration is very low. However, in the liver, PME concentration is about 1–3 mM [9,19,45], and the PME signal is one of dominant signals in the spectra.

#### 3.2.2. Phosphoenolpyruvate (PEP) and Phosphatidylcholine (PtdC)

The phosphoenolpyruvate (PEP) signal is a singlet and, in our phantom, showed a chemical shift between 2.0 and 2.27 ppm at a pH of 7.0 and 7.5, respectively. The strong dependence of the PEP signal on pH has already been described by Bierwagen [5], who measured chemical shifts of 1.53 and 2.13 ppm at a pH of 7 and 8, respectively. Similarly, Chmelik [4] reports a change in the chemical shift of PEP from −1 to 2.5 ppm in the pH range from 0 to 14 (~1.6 ppm at pH 7).

Very close to the chemical shift of PEP is the chemical shift of phosphatidylcholine (PtdC). It is one of the main compounds in the bile and can decrease in patients with cholangiopathies [46]. PtdC itself is very poorly soluble in water, and therefore, its resonance frequencies were obtained in ethanol–water mixtures (with a Triton−100 surfactant). We measured the chemical shift values between 2.14 and 2.13 ppm at pH 7.21 and 7.5, respectively. This is in accordance with the values obtained by Bierwagen [5] (chemical shift of PtdC of 2.15 and 2.16 ppm at a pH of 7 and 8) and Chmelik [4] (the phantom chemical shift of 2 ppm).

In the in vivo ^31^P MR spectra of the liver, the signal at 2.06 ppm was attributed to the combined signal of phosphoenolpyruvate and phosphatidylcholine, with an average concentration of 1.67 mM. Detailed studies [4,5] have shown that the main contribution comes from PtdC. In the liver parenchyma, this signal also appears and increases with decreasing distance of the VOI from the gallbladder. Therefore, the VOI for measuring the liver parenchyma should be carefully positioned to avoid unwanted signal overlap. In muscles in vivo, the PEP signal is at the noise level, so it cannot be evaluated and compared with the in vitro signal.

#### 3.2.3. Glucose-1-Phosphate (G1P) and Glucose-6-Phosphate (G6P)

Signals of both these compounds can be found in the range of 4 to 7 ppm. The G6P chemical shift position in vitro (7.03 ppm at pH = 7) corresponds to the in vivo position of G6P = 7.1–7.2 ppm [36,47].

Without proton decoupling G1P forms a doublet and G6P forms a triplet signal. The proton decoupling technique simplifies both of them to singlet signals. In the G6P signal, two other minor signals were found, probably from other enantiomer/isomers of glucose. A pH change from 7 to 7.5 results in a chemical shift difference of ±0.2 ppm for both metabolites.

In vivo skeletal muscle concentrations of G6P were shown to be around 0.1 (mg/kg muscle) [48,49] with a blunted, insulin-stimulated increase in insulin-resistant and type 2 diabetic patients, as well as in conditions in which plasma free fatty acids are elevated [40,50]. The muscle concentration of G1P is too low to be detected by ^31^P MRS in vivo; moreover, its signals are overlapped by other phosphomonoesters (PME) [47].

### 3.3. Phosphodiesters (PDE)

Phosphodiesters resonate in a frequency range from 2 to 4 ppm and include several compounds that are difficult to separate due to insufficient resolution if a low magnetic field is used. PDE may reflect (and serve as a marker of) membrane damage. Importantly, PME and PDE signals of ^31^P MRS may be also used in the diagnosis of liver diseases. In cirrhosis, the PDE signal has been shown to decrease compared to healthy controls or patients with steatosis [9,50].

The overall PDE concentration in the liver reported from ^31^P MR spectra in vivo is in the range between 5 and 13 mM [9,19,51,52,53,54,55,56]. It depends on the number of metabolites included, spectra resolution (1.5–7 T), and corrections to relaxation times.

PDE concentration in resting muscles ranges between 2–10 mM [11,52] and is related to energy metabolism [7] or neuromuscular conditions [57]. It increases with age [58] and in overweight subjects with a sedentary lifestyle [11] and also depends on physical status [59].

#### 3.3.1. Glycerophosphocholine (GPC)

Glycerophosphocholine (GPC) is, in addition to glycerophosphoethanolamine (GPE), the main component of the PDE signal observed in vivo. In the liver and muscles, GPC concentration is about 2–4 mM [4,11,19,56]. Recently, skeletal muscle and hepatic GPC were suggested as biomarkers of the tissue-specific action of thyroid hormones [10].

The chemical shift position of GPC was almost independent of pH. Proton decoupling simplified the triplet signal to a singlet. An in vitro chemical shift of 3 ppm was slightly different from the in vivo position of 2.76 ppm [4].

#### 3.3.2. 2,3-Diphosphoglycerate (2,3-DPG)

In the structure of 2,3-DPG, there are two phosphorus atoms providing signal—a doublet and a triplet at positions 4.1, 5.3 ppm (pH = 7) and 4.6, and 5.8 ppm (pH = 7.5) in a phantom. Proton decoupling simplifies this multiple structure to two singlets. In blood in vivo, both signals of 2,3-DPG are singlets at positions of 5.5 and 6.3 ppm. The difference between 2,3-DPG signals in vitro in a phantom and in blood may be explained by the 2,3-DPG association with hemoglobin in erythrocytes and its paramagnetic effect that strongly influences the chemical shift of 2,3-DPG [29].

The concentration of 2,3-DPG is strongly dependent on pH and very sensitive to the energy demand of the erythrocytes. The normal concentration of 2,3-DPG is about 4.5–5.1 mM packed red cells or 10.5–16.2 µmol/g Hb. Neonatal values are about 20% lower than those in an adult [60].

The presence of 2,3-DPG in erythrocytes is the basis for a typical ^31^P MRS signal of blood. Its presence in the ^31^P MR spectra of the heart indicates contamination of the myocardial signal by the signal of blood.

### 3.4. Nicotinamide Adenine Dinucleotide Metabolites (NAD^+^, NADH)

The pair of NAD^+^/NADH is an electron acceptor/donor in a redox reaction involved in cellular metabolism. The ^31^P signals of both compounds without ^1^H decoupling are singlets with positions at 8.32 (NAD^+^) and 8.16 (NADH) ppm, both insensitive to pH change. ^1^H decoupling reshapes the NAD^+^ signal to a doublet centered at 8.32 ppm, while the NADH signal only narrows. Measured in vitro shift positions are in good agreement with other in vitro and in vivo NAD^+^/NADH measurements, where shifts of ~8.1 ppm for NADH and ~8.3 ppm for the center of NAD^+^ have been reported with 4 to 11.7 T [61,62]. Similarly, in decoupled spectra, the NADH signal had the shape of a singlet and NAD^+^ had the shape of a doublet/quartet.

The concentration of NAD^+^/NADH and their ratio can be determined in vivo by MR techniques at magnetic fields of 3–7 T. However, the resonances of NAD^+^ and NADH are difficult to analyze separately without ^1^H decoupling. The analysis allows for the determination of the NAD^+^/NADH redox state of the tissue, e.g., in the brain, equal to 5.7 [21]. Total intracellular NAD^+^/NADH concentration is approximately 0.4 mM in the human brain [20,63] or 0.5 mM in the muscle of young, healthy volunteers [64].

### 3.5. Uridine Diphosphoglucose (UDPG)

The UDPG molecule contains two nonequivalent phosphorus nuclei. The J_P-P,_ as well as the J_H-P_ interaction, strongly modulates their signal. The application of proton decoupling simplifies the signal to two doublets with center positions at −9.8 ppm (phosphorus attached to the glucose part) and −8.2 ppm (phosphorus attached to the ribose part) [20,65]. In vivo, the UDPG signal at −8.1 ppm partially overlaps with NADH (resonating at −8.16 ppm), but signal at the −9.8 ppm position is well-resolved. The concentration of UDPG is approximately 0.2–0.3 mM in the brain [20] and 2.00  ±  0.22 mM wet tissue in the liver [19]. It seems that hepatic UDPG content does not change with age or weight [55], NAFLD, or cirrhosis [6,19].

Uridine diphosphate glucose (UDP-glucose) is an “activated” form of glucose because UDP provides additional energy to the glucose molecule. As such, UDPG is a precursor of polysaccharides (glycogen), glycoproteins, lipopolysaccharides, and glycosphingolipids. UDP-glucose can also be converted into UDP-galactose and UDP-glucuronic acid used in the synthesis of polysaccharides containing galactose and glucuronic acid.

### 3.6. ^31^P Basis Set for LCModel Calculations

The commonly used quantification of in vivo MR spectra is performed in the time domain using the jMRUI software package [66], or, in the frequency domain using LCModel software [14]. Both approaches require prior knowledge of chemical shifts, interaction constants, widths, and relative signal intensities within one molecule. These parameters are obtained by an analysis of the spectra of metabolites measured in vitro or from simulated spectra. The LCModel package is mostly used to analyze ^1^H in vivo MR spectra in the range of chemical shifts of 5–10 ppm. In this report, we used it to evaluate ^31^P in vivo spectra. Its application for the analysis of ^31^P spectra with signals resonating in the spectral range of about 30 ppm is more difficult and is not often used. Recently, however, a successful modification of the input parameters of the calculation of ^31^P and ^13^C spectra was proposed [16,67,68,69], which enabled the correct definition of the baseline of the spectrum. We adapted these input parameters, and we used our basis set for the analysis of ^31^P spectra of muscles and the liver. The chemical shifts of all 15 metabolites in phantoms measured at 3 T were in agreement with published high-resolution in vitro NMR spectra and simulated datasets. In addition, small changes of chemical shifts of ATP were necessary for in vivo applications, as described above. Two other signals were added to the BASIS^p^—the signal of membrane phospholipids and GPE based on the parameters from the literature [15]. The basis set used for the analyses contained 19 signals of 18 model spectra (Table 1)

The full BASIS^p^ set for LCModel was used in a representative calculation of the ^31^P MR calf muscle and liver spectra, as shown in Figure 1.

In addition, the comparison of the LCModel and jMRUI methods was performed on a group of five randomly selected ^31^P liver spectra and five spectra from the calf muscle (at rest) from our internal database, see Table 2. The modified BASIS^p^ set with only six prominent signals in both measured tissues was used. The calculated relative intensities are similar for most signals, as shown by the coefficients of variation (CV) of the metabolite relative intensity signals in Table 2. Concerning muscle spectra, both methods give similar results with the highest but still acceptable CV for GPC and Pi. As expected in the liver due to lower signal to noise, the highest CV values (CV > 30, unacceptable difference) were obtained for GPC signal, which represents different PDE metabolites. In addition, PCr signal was also used in this case for comparison, although it represents only contamination from the surrounding tissue, see Table 2. CVs in this pilot test show that both LCModel and jMRUI methods often reach similar results; the difference originates from different mathematical principles of signal intensity calculation, as described in the original literature [14,66].

Regarding the calculation of the concentration of metabolites, a number of papers have been published concerning the application of LCModel frequency domain approach to 1H MRS (mostly brain), and most of the 31P MRS reports used the AMARES based jMRUI time–domain fitting approach. Besides the historical reason for this difference, there was no available complete basis set for muscle or liver 31P MRS that could be used for LCModel. In line with our experience from 1H MRS data processing, the LCModel is more useful for routine calculation of larger data sets than jMRUI. The AMARES-based jMRUI approach, on the other hand, can be used more easily to characterize and analyze a single spectrum. Based on our experience and the result of this study, we think that it is useful to combine both methods for evaluating spectra.

## 4. Materials and Methods

### 4.1. Phantom Preparation

All chemicals from Table 1 were purchased from the Sigma-Aldrich Company. Structural formulas are depicted in Supplementary Materials. MgCl_2_, K_2_HPO_4_, and NaH_2_PO_4_ were obtained from Lachema, Brno, Czech Republic.

Phantoms of 100 mL volume consisted of water solutions: MgCl_2_ (concentration C_MgCl2_ = 0.35 mM), K_2_HPO_4_, and NaH_2_PO_4_ 1:1 (concentration C_sum_ = 40 mM) and phosphocreatine (concentration C_PCr_ = 5 mM) (as a standard). To this solution, metabolites were added to reach a final concentration of 10 mM; see details in Table 1. UDPG and 2,3-DPG phantoms were prepared with a lower concentration of c ~2 mM. In these phantoms, the concentration of K_2_HPO_4_/NaH_2_PO_4_ was reduced to c = 10 mM.

MgCl_2_ was added to phantoms to stabilize solutions because Mg^2+^ ions often form complexes with ^31^P metabolites that may affect the shape and chemical shift of the ^31^P metabolites [37,70]. The K_2_HPO_4_ and NaH_2_PO_4_ were used to buffer pH and prevent cleavage of Pi from ^31^P metabolites.

Two phantoms with a pH of 7.03 and 7.50 were prepared for each metabolite. pH was adjusted by titration of NAOH/HCl in solutions at 37 °C and slightly fluctuated about ±0.05 during adjustment. The pH of phantoms was also verified during MR measurement from differences in the chemical shift between the PCr and Pi signal.

In addition, a blood phantom for in vitro measurement of human venous blood was prepared for the comparison of 2,3-DPG solutions. The blood phantom consisted of 50 mL human blood with heparin.

Two phantoms were prepared without other specific metabolites containing PCr (C_PCr_ = 5 mM), MgCl_2_, and K_2_HPO_4_/NaH_2_PO_4_ with a pH = 7.03 and 7.5 to test the pH dependency of the chemical shift position of PCr on pH.

### 4.2. ^31^P MR Spectroscopy

**Figure 2:**
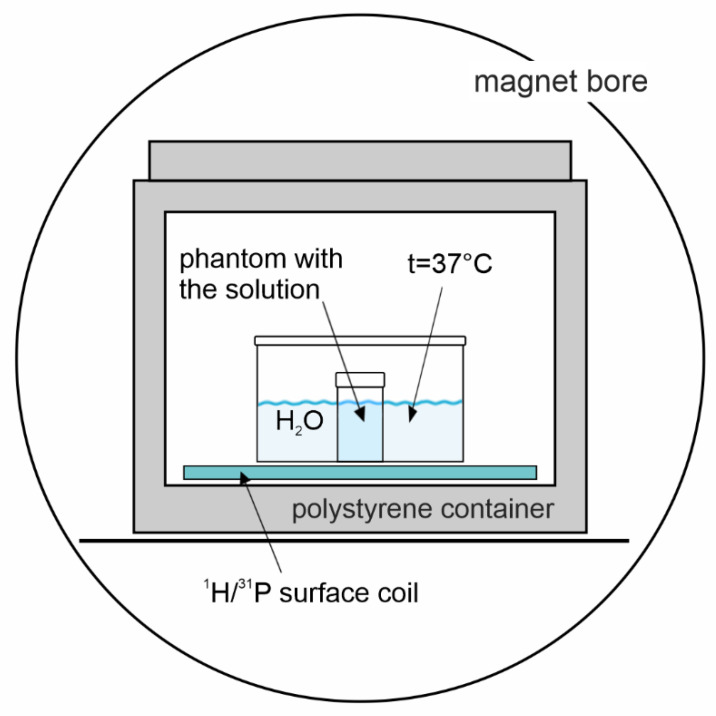
Arrangement of the phantom ^31^P MRS experiment. A plastic bucket filled with water keeps the temperature and loading of the coil.

Measurements were performed using a 3T MR system, VIDA (Siemens Healthineers, Erlangen, Germany) equipped with a dual-channel ^1^H/^31^P surface coil (Rapid Biomedical, Rimpar, Germany) with a diameter of 11 cm and an interface device; see Figure 2.

An MRI localizer sequence was used to verify the position of the phantom and adjustment volume, which was defined as a cube (35 × 35 × 35 mm) in the center of the phantom. Automatic and manual adjustment of magnetic field homogeneity were performed at the water signal; an excellent half-width of the water signal (3–4 Hz) was achieved for all phantoms in defined adjustment volume (a critical step in achieving excellent magnetic field homogeneity was to align the water level in the phantom and temperature reservoir to the same position before measurement).

Two free induction decay (FID)-based non-localized ^31^P MR spectroscopic sequences were applied in each phantom with the same parameters, as follows: repetition time (TR) of 15 s; acquisition delay (TE*) = 0.4 ms, number of acquisitions (acq) = 64; flip angle (FA) = 90° (BIR-4 adiabatic pulse); vector size = 2048; and bandwidth = 1000 Hz (20 ppm) to cover chemical shift of studied metabolites. Transmitter frequency was set to that of the PCr signal except for the ATP measurement; it was shifted to the signal of αATP in this case to excite all ATP signals sufficiently. Non-slice selective proton decoupling (the WALTZ4 decoupling scheme) was applied in the second sequence to cancel the J_P-H_ interaction (standard Siemens setting—decoupling pulse duration 1 ms, decoupling duration 1000 ms, decoupling flip angle 90 or 180°, offset frequency for 1H—0 ppm, duty cycle 50%).

### 4.3. Spectra Evaluation

Spectra were loaded into jMRUI software (version 6.0). Manual correction of the phase (zero- and first-order) was performed to obtain the absorption shape of phosphorous signals, and then the position of the PCr and second metabolite were read according to their maxima. The BASIS^p^ set for ^31^P LCModel version 6.3–0 G was constructed from phantom chemical shifts presented in Table 1.

To analyze in vivo data, the spectra were phased manually in the jMRUI software to obtain reliable prior knowledge of the zero- and first-order phase parameters used further in the LCModel analysis (starting points and standard deviations, DEGZER = −96, SDDEGZ = 10, DEGPPM = 7, SDDEGP = 4). In addition, the following LCModel input parameters were used: DKNTMN = 2*99; XSTEP = 5; RFWHM = 3; FWHMBA = 0.049; NREFPK(2) = 1; PPMREF(1,2) = 0; and DESDSH = 0.01. The default values that define the spline baseline function were also adjusted to take into account the larger spectral range of ^31^P compared to ^1^H: ALPBMN = 7.8 × 10^−10^; ALPBMX = 3.9 × 10^−7^; ALPBPN = 9.8 × 10^−10^; and ALPBST = 1.2 × 10^−9^, as mentioned previously [71]. A representative evaluation of in vivo ^31^P MR spectra of the liver and calf muscles using LCModel software and the BASIS^p^ basis set is shown in Figure 1 and Table 2.

## 5. Conclusions

The ^31^P chemical shifts of 15 metabolites were measured under conditions close to those of an in vivo examination at 3T and were compared and supplemented with published values for in vitro and in vivo conditions. Based on all these data, a set of 19 signals necessary for the successful quantification of in vivo liver and muscle spectra by LCModel software was generated. The tabulated values of chemical shifts can also be used for other methods of in vivo spectra analysis, e.g., jMRUI software and other analytical procedures.

## Figures and Tables

**Table 1:** Concentration of ^31^P MR phantoms, their chemical shifts (ppm) and coupling constants.

Metabolite	Product Number		Concentration in Phantoms (mM)	Multiplicity	Chemical Shift at pH = 7.0	Chemical Shift at pH = 7.5	Chemical Shift In Vivo	J-Coupling (Hz)	Basis Set Deelchand	BASIS^p^ Set Present Study
**Phosphocreatine**	P7936-5G	^1^ PCr	5	s	0	0	0	-	0	**0**
**Inorganic phosphate**		Pi	40	s	4.78	5.27	~5	-	4.84	**4.78**
**Adenosine triphosphate**	A2383-1G	αATP	10	d	−7.98	−7.94	~7–8	19.5	−7.56	**−7.53**
		βATP	10	t	−18.80	−18.58	~16	20.0	−16.18	**−16.18**
		γATP	10	d	−4.18	−3.37	~3–4	19.0	−2.53	**−2.7**
**Adenosine diphosphate**	29349990900	αADP	10	d	−7.28	−7.32	-	19.0	-	**-**
		βADP	10	d	−3.50	−3.20	-	19.5	-	**-**
**Phosphoethanolamine**	P0503-1G	PE (PME)	10	t	6.74	6.85	6.78	7.0	6.77	**6.77**
**Phospholcholine**	P0378G	PC (PME)	10	t	6.19	6.35	5.9	6.0	6.23	**6.23**
**Glucose 1-phosphate**	G1259-1G	G1P	10	d	4.99	overlay with Pi (5.20)	overlay with PME	7.5	-	**4.99**
**Glucose 6-phosphate**	G7879-1G	G6P	10	t	7.03	7.30	7.1–7.2	6.1	-	**7.03**
**Phosphoenolpyruvate**	P7127-500MG	PEP (PME)	10	s	2.00	2.27	2.06		-	**2**
**Phosphatidylcholine**		PtdC	^3^ 10	t	2.14	2.13	2.06		-	**2.14**
**Glycerol-3-phosphoryl choline**	G5291-100MG	GPC (PDE)	10	t	2.97	2.96	2.76	5.5	2.94	**2.97**
**2.3-diphosphoglycerate**	D9134-100MG	2,3-DPG(PDE)	^2^ 2	2- d	4.09	4.6	5.5	6.0	5.23	**4.05**
				3- t	5.338	5.84	6.3	9.5	5.71	**5.32**
**Nicotinamide adenine dinucleotide (reduced form)**	N8129-1G	NADH	10	s	−8.16	−8.16	~8.1		−8.13	**−8.13**
**Nicotinamide adenine dinucleotide (oxygenated form)**	10127981001	NAD^+^	10	s	−8.32	−8.32	~8.3	6.0	−8.31	**−8.31**
**Uridine diphosphoglucose**	U4625-500MG	UDPG	^2^ 2	d	−8.11	−8.11	−8.1	10.0	-	**−7.98**
		UDPG	^2^ 2	d	−9.78	−9.78	−9.8	10.0	-	**−9.78**
**^4^** **Membrane phospholipids**		MP							2.3	**2.3**
**^4^** **Glycerophosphoethanolamine**		GPE							3.49	**3.49**

^1^ PCr was used as an internal standard; ^2^ K_2_HPO_4_/NaH_2_PO_4_ to c = 10 mM; ^3^ 40% ethanol with the addition of Triton 100 as a surfactant; ^4^ taken from [15]. s—singlet; d—doublet; t—triplet.

**Table 2:** Pilot comparison of the LCModel and jMRUI method calculation of means of relative signal intensities (%) and relative Cramér Rao Lower Bounds (CRLB, %) of five liver and muscle spectra and means of their coefficients of variance CV (%) for the comparison. The BASIS^p^ set was applied.

**Liver**		**γ-ATP**	**α-ATP**	**β-ATP**	**GPC**	**PCr**	**Pi**
jMRUI	Relative signal intensity	23.3	26.9	13.4	21.6	2.5	12.3
	Relative CRLB	2.0	1.9	4.0	3.9	19.6	3.9
LCModel	Relative signal intensity	26.0	34.1	11.3	15.6	3.3	9.7
	Relative CRLB	3.3	2.7	4.7	6.0	11.0	5.0
mean CV		11	23	16	37	33	23
**Muscle**		**γ-ATP**	**α-ATP**	**β-ATP**	**GPC**	**PCr**	**Pi**
jMRUI	Relative signal intensity	12.4	10.3	10.0	2.6	60.2	4.4
	Relative CRLB	1.2	1.5	2.0	8.8	<1	1.9
LCModel	Relative signal intensity	14.6	12.2	10.5	3.4	53.6	5.7
	Relative CRLB	1.7	2.0	2.3	8.7	<1	2.0
mean CV		16	17	5	24	12	25

The characterization of CV was used: CV < 10 is very good agreement, 10–20 is good agreement, 20–30 is acceptable agreement, and CV > 30 is not acceptable.

## Data Availability

On demand to: petr.sedivy@ikem.cz, monika.dezortova@ikem.cz.

## Supplementary Materials

The following are available online: spectra and chemical structures of measured metabolites.

## Sample Availability

Samples of the compounds are not available from the authors.
