# Supplementary material for: In Vitro 31P MR Chemical Shifts of In Vivo-Detectable Metabolites at 3T as a Basis Set for a Pilot Evaluation of Skeletal Muscle and Liver 31P Spectra with LCModel Software
Source: Molecules. 2021 Dec 14;26(24):7571. doi: 10.3390/molecules26247571 (PMC8703310; doi:10.3390/molecules26247571)

Supplementary Materials: spectra and chemical structures of measured metabolites

^{31}P MR spectra of all described metabolites in phantom solution measured at 3T. The measurement temperature was 37°C and the pH from 7.0 to 7.5. Details are given in the methods in the text. The phosphocreatine (PCr) signal was used as standard with a chemical shift of 0 ppm. Spectra were measured without (red lines) and with ^{1}H decoupling (blue lines).

Pi, inorganic phosphate

PCr, Phosphocreatine
N-Methyl-*N*-(phosphonocarbamimidoyl)glycin[illegible]

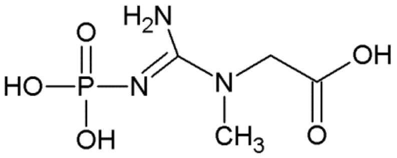

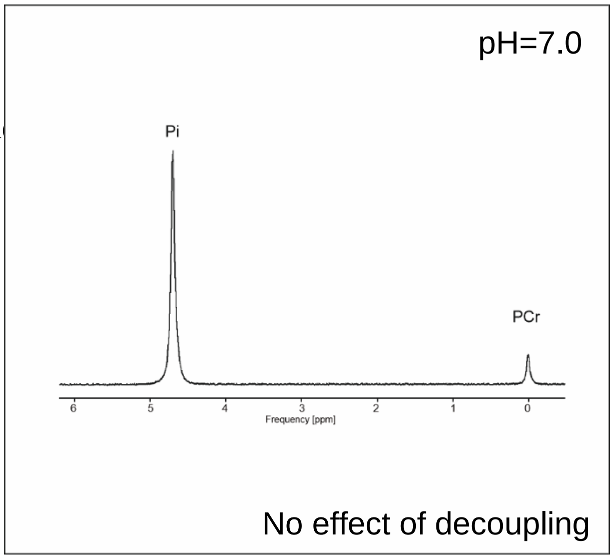

NAD^{+}, NADH
Nicotinamide adenine dinucleotide
Diphosphopyridine nucleotide (DPN^{+}),
Coenzyme I

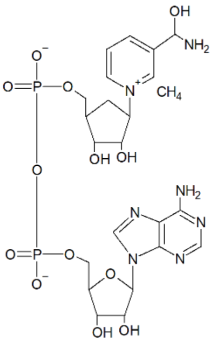

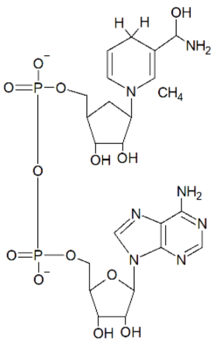

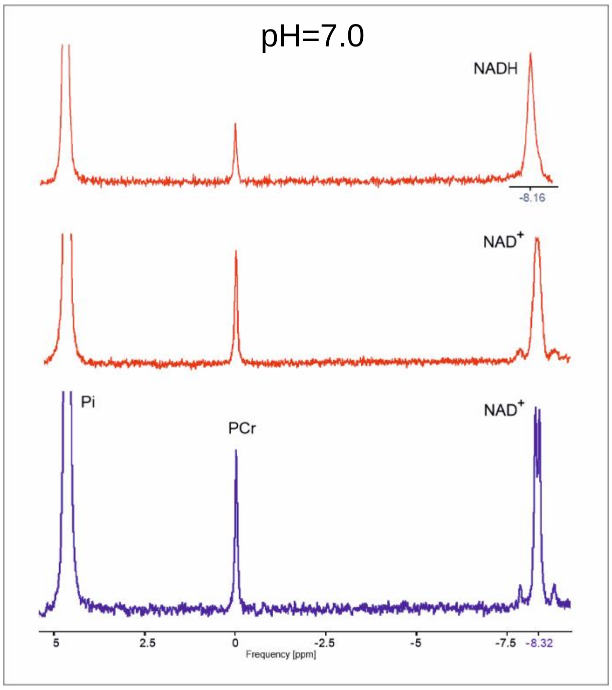

PEP, Phosphoenolpyruvate
2-(Phosphonooxy)prop-2enoic acid

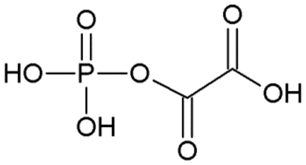

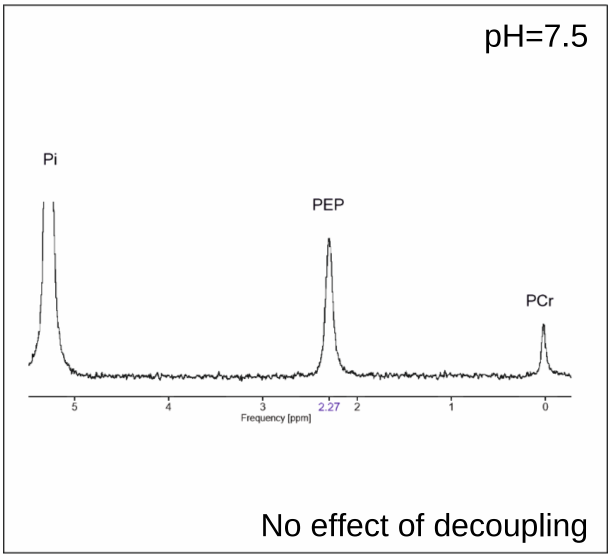

UDPG, Uridine diphosphate glucose
O1-{[(2R,3S,4R,5R)-5-(2,4-Dioxo-3,4-dihydropyrimidin-1(2H)-yl)-3,4-dihydroxyoxolan-2-yl]methyl} O3-[(2R,3R,4S,5S,6R)-3,4,5-trihydroxy-6-(hydroxymethyl)oxan-2-yl] dihydrogen diphosphate

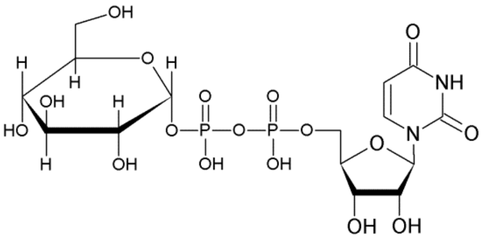

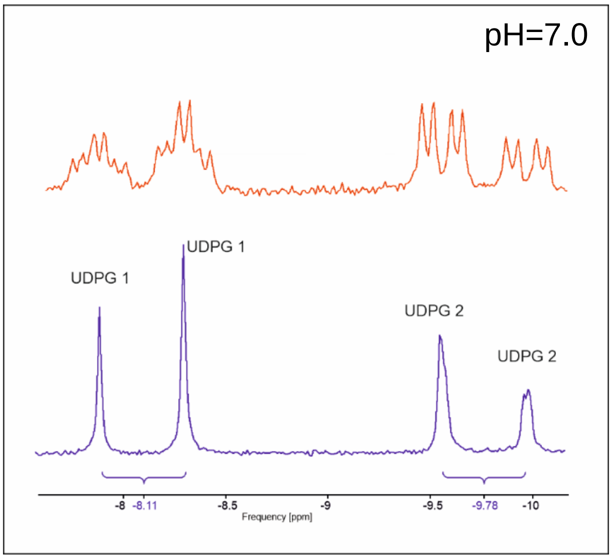

G1P, Glucose 1-phosphate
(2*R*,3*R*,4*S*,5*S*,6*R*)-3,4,5-Trihydroxy-6-(hydroxymethyl)oxan-2-yl phosphate

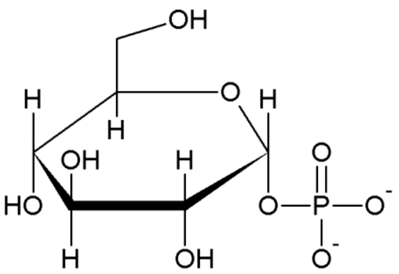

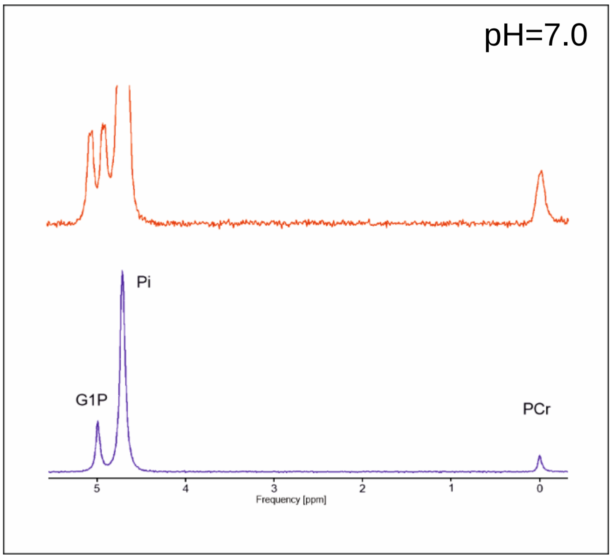

G6P, Glucose 6-phosphate

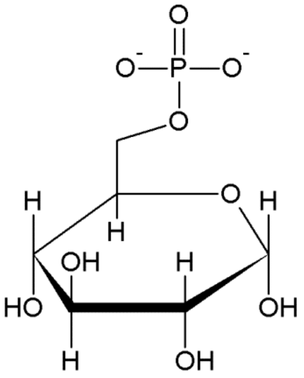

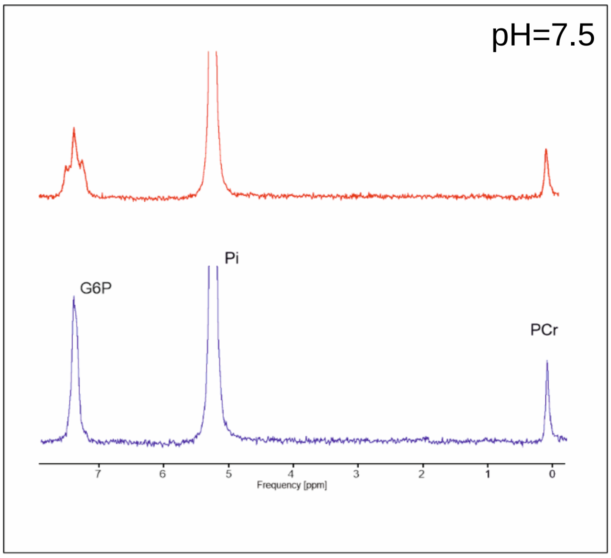

PC Phosphocholine
Choline phosphate

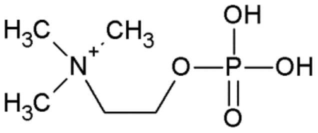

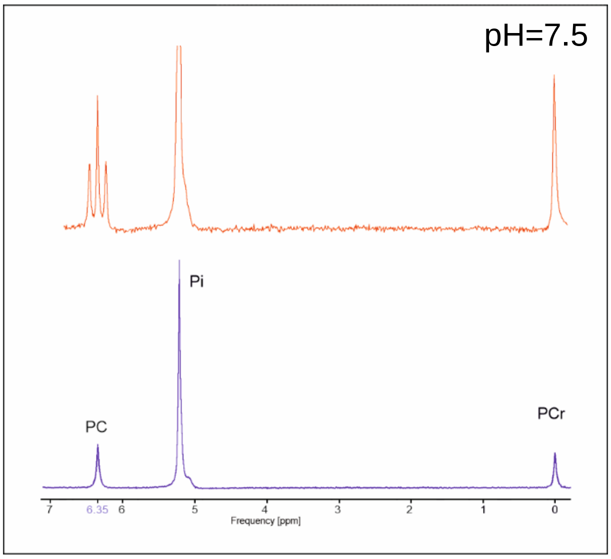

PE Phosphorylethanolamine
Phosphoethanolamine
2-Aminoethyl dihydrogen phosphate

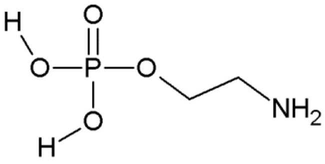

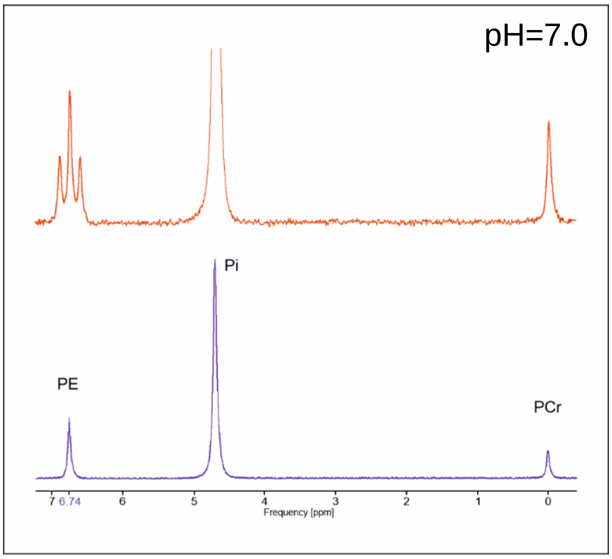

GPC
Glycerol-3-phosphoryl choline

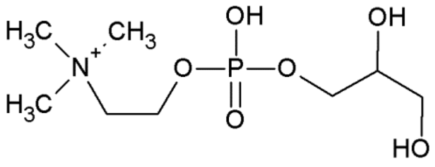

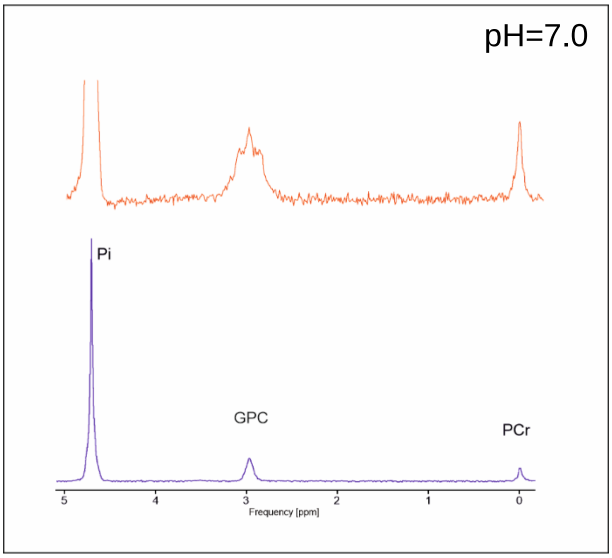

ATP, Adenosine triphosphate
Adenosine 5′-(tetrahydrogen triphosphate)

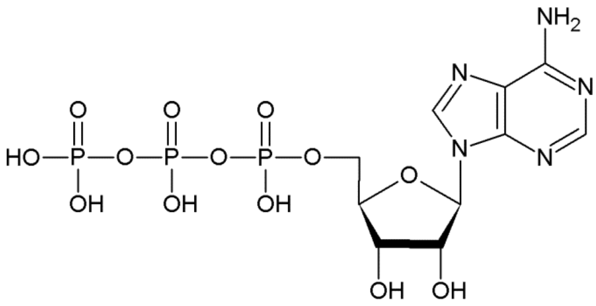

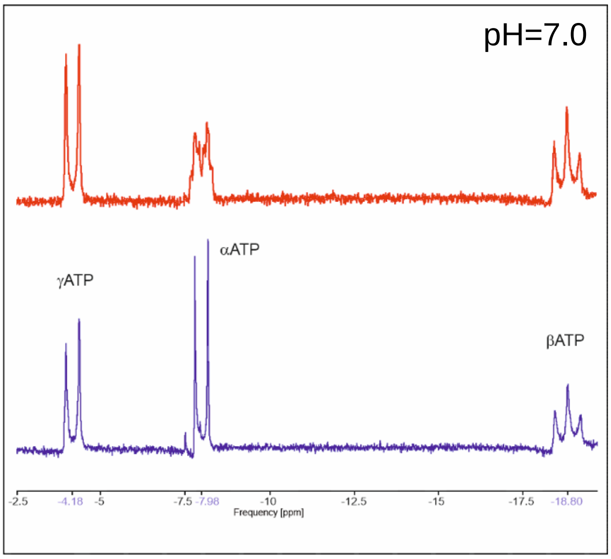

ADP, Adenosine diphosphate
Adenosine 5′-(trihydrogen diphosphate)

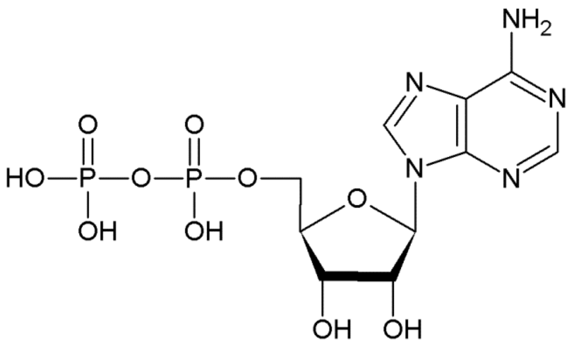

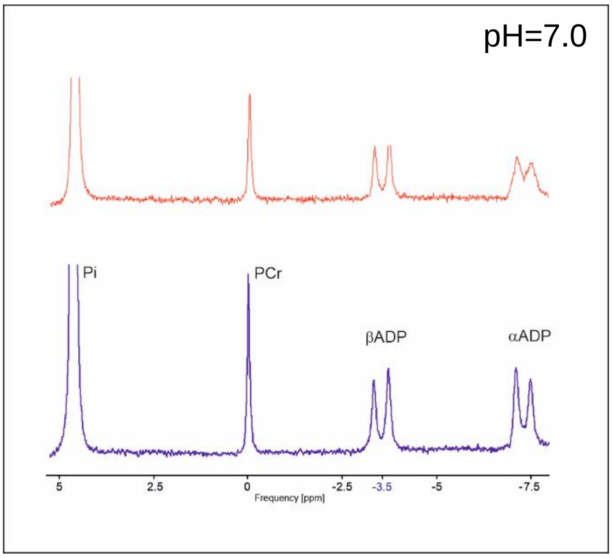

2,3-DPG, 2,3-diphosphoglycerate)
2,3-Bis(phosphonooxy)propanoic acid

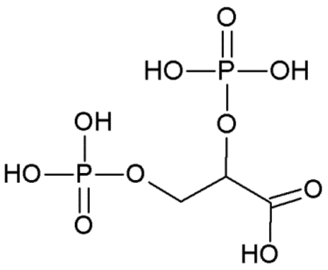

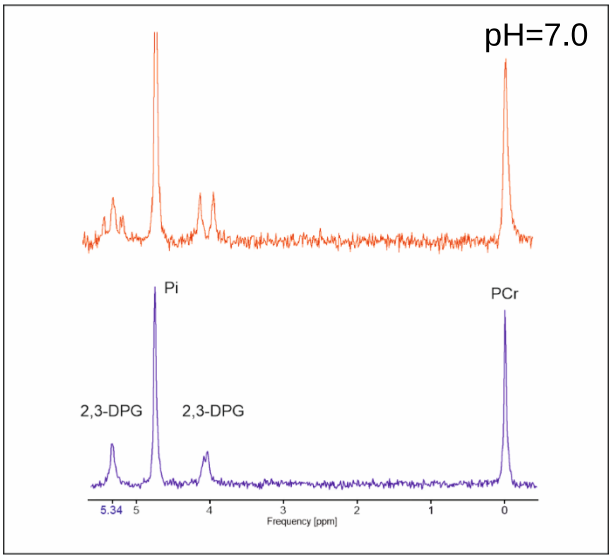

PtdC, Phosphatidylcholines
1-Oleoyl-2-palmitoyl-phosphatidylcholine
(solution of water+ethanol+tritone)
*impurities

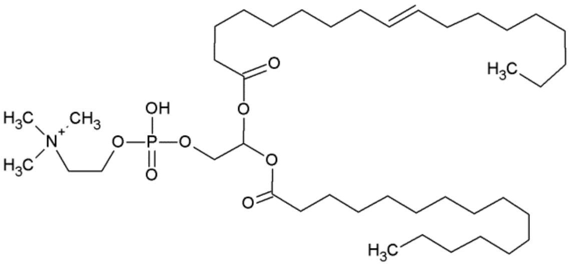

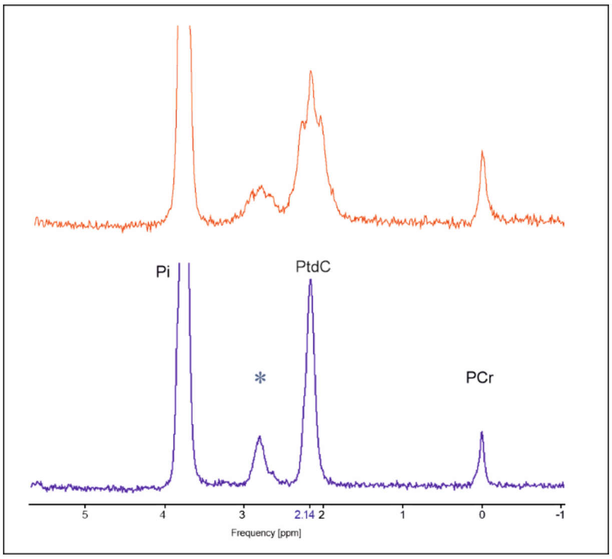

Supplement: Supplementary file 1 [file molecules-26-07571-s001.zip › molecules-1395164-supplementary.pdf]
